# Uncovering the Oral Dysbiotic Microbiota as Masters of Neutrophil Responses in the Pathobiology of Periodontitis

**DOI:** 10.3389/fmicb.2021.729717

**Published:** 2021-10-11

**Authors:** Zsombor Prucsi, Alicja Płonczyńska, Jan Potempa, Maja Sochalska

**Affiliations:** ^1^Department of Microbiology, Faculty of Biochemistry, Biophysics and Biotechnology, Jagiellonian University, Krakow, Poland; ^2^Department of Oral Immunity and Infectious Diseases, University of Louisville School of Dentistry, Louisville, KY, United States

**Keywords:** periodontitis, neutrophils (PMNs), innate immunity, virulence factor, inflammation

## Abstract

Numerous bacterial species participate in the shift of the oral microbiome from beneficial to dysbiotic. The biggest challenge lying ahead of microbiologists, immunologists and dentists is the fact that the bacterial species act differently, although usually synergistically, on the host immune cells, including neutrophils, and on the surrounding tissues, making the investigation of single factors challenging. As biofilm is a complex community, the members interact with each other, which can be a key issue in future studies designed to develop effective treatments. To understand how a patient gets to the stage of the late-onset (previously termed chronic) periodontitis or develops other, in some cases life-threatening, diseases, it is crucial to identify the microbial composition of the biofilm and the mechanisms behind its pathogenicity. The members of the red complex (*Porphyromonas gingivalis*, *Treponema denticola*, and *Tannerella forsythia*) have long been associated as the cause of periodontitis and stayed in the focus of research. However, novel techniques, such as 16S clonal analysis, demonstrated that the oral microbiome diversity is greater than ever expected and it opened a new era in periodontal research. This review aims to summarize the current knowledge concerning bacterial participation beyond *P. gingivalis* and the red complex in periodontal inflammation mediated by neutrophils and to spread awareness about the associated diseases and pathological conditions.

## Biofilm Impact on Neutrophils in the Development of the Periodontal Disease

**FIGURE 1:**
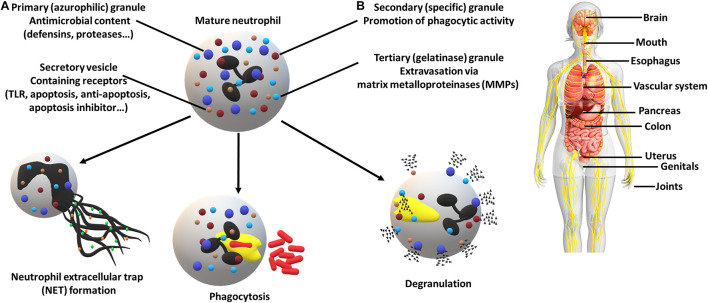
Neutrophil defense mechanisms **(A)** and associated diseases **(B)**. **(A)** Neutrophils can employ numerous strategies in order to eliminate pathogens, including the formation of neutrophil extracellular traps, destined to capture and eliminate, internalization (phagocytosis) and the release of a high variety of granule contents (e.g., receptors, proteases and enzymes). The utilization of secretory vesicles provides an easy to mobilize source of receptors crucial for pathogen recognition (TLRs) and cell fate determination (apoptosis regulators). **(B)** Periodontal pathogens have been associated with multiple other diseases which resulted in increased attention. Affected organs and systems (non-exhaustive): brain (Alzheimer’s disease), mouth (periodontitis, oral squamous cell carcinoma, peri-implantitis), esophagus (esophageal cancer), vascular system (aortitis, atherosclerosis), pancreas (pancreatic cancer), colon (colorectal cancer), uterus (preterm birth), genitals (bacterial vaginosis), joints (rheumatoid arthritis).

The innate immune system is the first line of defense against pathogenic invasion. The response begins with the recruitment of immune cells. In the oral cavity, the most abundant contributors are neutrophils. The mechanism of the immune system involves the promotion of inflammation, recruitment of other immune cell types and use of neutrophil-specific defense mechanisms (Figure 1). The coordinated attack against pathogens involves the formation of Neutrophil extracellular traps (NETs), a web-like structure destined to capture and eliminate, the internalization (a.k.a. phagocytosis) and the release of the diverse granule content (Scott and Krauss, 2012; Vladimer et al., 2013; Li et al., 2020). In response to the biofilm microbiome, e.g., Fusobacterium nucleatum a significant change in neutrophil gene expression is observed (Wright et al., 2011).

Within the oral biofilm, pathogens have developed countless sophisticated strategies to bypass elimination and turn an inflamed environment in their favor, such as manipulation of neutrophil survival, prolonged inflammatory responses or subversion of anti-microbial properties (White et al., 2014; Olsen and Yilmaz, 2016). Throughout the shift of the microbial composition of the oral cavity, also known as the development of periodontitis, some pathogens act as bridging species between early and late contributors. During the progression of the infection, a strong manipulation of the neutrophil function by the biofilm pathogens can be identified (Table 1). Research-wise, it is highly challenging to reveal the interaction between multi-species biofilms and neutrophils. The first step is to identify pathogen-specific effects.

**TABLE 1:** Identified prevalent pathogens associated with periodontitis (non-exhaustive).

Name & Gram staining [+/*−*]	Use of oxygen	Virulence factor	Effect on neutrophil function	Association with other disease	Sources
*Porphyromonas gingivalis* −	Anaerobic	Gingipain	Abolish pro-inflammatory signaling	Alzheimer’s disease	Popadiak et al., 2007; Ilievski et al., 2018
*Treponema denticola* −	Anaerobic	Major outer sheath protein (Msp)	Distract neutrophil chemotaxis	Oral squamous cell carcinoma (OSCC)	Jones et al., 2017; Fitzsimonds et al., 2020
*Tannerella forsythia* −	Anaerobic	Miropin	Neutrophil protease inhibitor	Atherosclerotic lesions	Honma et al., 2009; Lee et al., 2014; Ksiazek et al., 2015
*Fusobacterium nucleatum* −	Anaerobic	Serine protease	Degrade extracellular matrix proteins	Colorectal cancer	Signat et al., 2011; Han, 2015
*Prevotella intermedia* −	Anaerobic	nucA/D	Degrade neutrophil extracellular traps (NETs)	Aortitis	Nambu et al., 2015; Boersma et al., 2017; Doke et al., 2017
*Aggregatibacter actinomycetemcomitans* −	Anaerobic	Extracellular adenosine triphosphate (eATP)	Recruitment of immune cells	Rheumatoid arthritis	Ding et al., 2016; Konig et al., 2016
*Peptoanaerobacter stomatis* +	Anaerobic	Neutrophil-derived chemokines	Induced chemotaxis of both neutrophils and monocytes.	Unknown	Sizova et al., 2015; Vashishta et al., 2019
*Filifactor alocis* +	Anaerobic	High tolerance against oxidative stress	Avoid oxygen-dependent defense mechanism	Peri-implantitis	Aruni et al., 2011, 2015

## Pathogen-Specific Effects on Neutrophil Functions

### Fusobacterium nucleatum

*Fusobacterium nucleatum* is recognized as a master species in the development of periodontitis, with many strain-specific functions. In order to enhance the multiplication of late colonizers, it is crucial to create favorable conditions with priority to decrease neutrophil efficiency. This is carried out by decreasing superoxide generation and apoptosis induction, limiting the number of counter-attacking immune cells (Kurgan et al., 2017). The production and release of reactive oxygen species (ROS) is part of the pathogen elimination strategies and can be induced by the phagocytosis of the invaders (El-Benna et al., 2016; Zeng et al., 2019). In contrast to well-characterized pathogens, the amount of *F. nucleatum* differs within the timeline of disease progression in accordance with its bridging-specie nature (Tomšič et al., 2021). Moreover, it has been proved that *F. nucleatum* exaggerates NET formation in comparison to other bacterial species, such as *P. gingivalis*, in strong connection with NOD-like receptors (Alyami et al., 2019). Triggering NETosis, instead of phagocytosis, as a neutrophil defense mechanism is probably associated with the huge size of the pathogen (Urban et al., 2006). Another antimicrobial mechanism strongly exploited by *F. nucleatum* is the release of Human Neutrophil Peptide-1 (HNP-1) (Musrati et al., 2016). Increased concentrations of HNP-1 peptide trigger epithelial cell death and bacterial attachment to keratinocytes (Gursoy et al., 2013). Exposure of *F. nucleatum* to defensins, a class of antimicrobial peptides released for instance by neutrophils, can result in decreased bacterial membrane permeability and elevated dental plaque biofilm formation. These strategies are destined to aid the resistance against the membrane disruption and lysis caused by the above-mentioned proteins. As a consequence, the proliferation level is secured (Keskin et al., 2014; Xu and Lu, 2020). Importantly, secreted serine protease fusolisin degrades extracellular matrix proteins as well as cleaves the most abundant immunoglobulin at the mucosal surface (IgA), contributing to the inactivation of the host defense and disease progression (Bachrach et al., 2004; Doron et al., 2014). To add insult to injury, observed tissue environment modulation can give rise to other pathological conditions, such as colorectal cancer (Luo et al., 2019).

### Prevotella intermedia and Tannerella forsythia

Pathogens can also have an indirect effect on the function of neutrophils. In the case of *Prevotella intermedia* and *Tannerella forsythia*, it has been proved that they can adhere to and internalize into human dental follicle stem cells (hDFSCs). This infection modulates the environment and diminishes the expression of cytokines, however, it does not change hDFSC differentiation capacity. Consequently, a reduced release of IL-8 can contribute to limited chemotaxis of polymorphonuclear leukocytes (PMNs). Moreover, in the presence of infected hDFSCs neutrophil phagocytic activity and NET formation are also decreased, which gives pathogens enough time for gingival colonization (Hieke et al., 2016). Importantly, a challenge with *T. forsythia* induces a strong immune response as indicated by the number of immune cells accumulated at the lesion of administration in a murine model. These *in vivo* experiments showed, that unlike in the presence of *P. gingivalis*, the neutrophil migration is not inhibited (Gosling et al., 2005). However, some comorbidity, such as glycogen storage diseases (GSDs), can in few cases further enhance gingival tissue destruction. A case report of a GSD patient manifesting with the subversion of the neutrophil chemotaxis and neutropenia described an evident *T. forsythia*-mediated intraoral bone loss (Ma et al., 2018).

Interestingly, Ksiazek et al. discovered that *T. forsythia* expresses a serpin (protease inhibitor) called miropin that can contribute to its survival and ability to avoid protease activity of neutrophils (Ksiazek et al., 2015). Strikingly, another newly discovered metalloproteinase called ***miro***lysin together with the previously characterized, secreted metalloproteinase called karilysin, represent important virulence factors of *T. forsythia*. Both proteinases show a synergistic inhibitory effect on many pathways in the host immune system. Significantly, *T. forsythia* with a mutation in the expression of these enzymes presented with a strongly diminished survival rate (Jusko et al., 2012, 2015). Apart from proteinases, an outer surface layer (S-layer) is a *T. forsythia*-associated virulence factor that can contribute to serum resistance and has a significant role in coaggregation with other oral pathogens, such as *P. gingivalis.* The S-layer significantly reduces the deposition of C3b on the bacterial surface, which would act as a tag for phagocytosis (Shimotahira et al., 2013). Strikingly, a bacterial glycan found linked to the S-layer can modulate dendritic cells and suppress T helper 17 response (Settem et al., 2013). As an anaerobic bacteria, *T. forsythia* lacks a complex enzymatic system against oxidative stress. However, the genome of this pathogen encodes an oxidative stress response sensor protein (OxyR) homolog, that acts as a positive regulator for antioxidant gene expression. This can contribute to the resistance of the bacterial community to oxidative stress in the aerobic oral cavity and protect against oxidative burst in leukocytes, which is essential in the dental plaque biofilm formation (Honma et al., 2009; Moriguchi et al., 2017).

The main component of NETs is DNA (Brinkmann et al., 2004). Among other species (*P. gingivalis*, *F. nucleatum* and *Aggregatibacter actinomycetemcomitans*), *P. intermedia* has the highest nuclease activity, enabling it to block the capture and subsequent phagocytosis by neutrophils. Two genes have been identified as responsible for this feature, *nucA* and *nucD*, encoding enzymes that require cations for their activity (Doke et al., 2017). Still, this strategy itself cannot be considered unique, as numerous pathogens associated with periodontal disease are able to express such enzymes, including members of the red and the orange complexes (Palmer et al., 2012). Bacteria species are grouped based on chronological coexistence during diseases progression. Members of the red complex (*Porphyromonas gingivalis*, *Treponema denticola* and *Tannerella forsythia*) are late colonizers and the multiplication of them relies on, and is tightly connected to the conquest of the members of the orange complex (e.g., *Fusobacterium nucleatum*, *Prevotella intermedia*) (Mohanty et al., 2019).

### Aggregatibacter actinomycetemcomitans

Secreted extracellular adenosine triphosphate (eATP) is a distinctive virulence factor characterized in *A. actinomycetemcomitans*. eATP is predominantly an intracellular signaling molecule involved in the recruitment of immune cells (Ding et al., 2016). However, in periodontitis, eATP secreted by *A. actinomycetemcomitans* induces an upregulation of cytokine expression, resulting in the massive recruitment of inflammatory cells via mainly the p38 mitogen-activated protein kinase (MAPK) and MAPK-activated protein kinase 2 (MK2) pathways (Herbert et al., 2017). Therefore, would be of great importance to investigate the effects of eATP on p38 or MK2 kinases not only in macrophages, but also in neutrophils Interestingly, macrophages challenge with *A. actinomycetemcomitans* induced autophagic influx, restricting the expression of the proinflammatory cytokine IL-1β and ROS production, which ensure protection for this pathogen (Lee et al., 2020). Of importance, bacterial metabolites, such as short-chain fatty acids (SCFAs), are widely spread among different species and connected to a great number of immunological disorders (Ferreira et al., 2014). During bacterial infection, *A. actinomycetemcomitans*-associated SCFAs can also alter neutrophil effector mechanisms by downregulating cytokine production and phagocytic activity (Corrêa et al., 2017).

Leukotoxins are virulence factors expressed e.g., by some *Staphylococcus spps*. or by *Mannheimia haemolytica*, altering both the innate and the adaptive immune system (Futagawa-Saito et al., 2004; Aulik et al., 2010). Strikingly, leukotoxin A (LtxA) secreted by *A. actinomycetemcomitans* triggers a dysregulation in neutrophils, resulting in the release of citrullinated proteins (Konig et al., 2016). The hypercitrullination of host proteins, on one hand leads to diminished functions, such as in the case of histone proteins embedded in NETs can be responsible for decreased anti-microbial activity (Li et al., 2010). On the other hand, the development of rheumatoid arthritis (RA) is the consequence of an elevated level of citrullinated proteins, that leads to the hyperactivity of the immune system resulting in the destruction of the host tissue (Kuhn et al., 2006). The link between RA and periodontitis has long been under the scope of scientific research due to the numerous shared immune-pathological similarities, including overall disease progression, cytokine profile and risk factors (Koziel et al., 2014; de Molon et al., 2019). As these two diseases can present with similar symptoms, some approaches can be applied in both cases to control disease manifestations, i.e., the melanocortin agonism can be a potential way to overcome excessive oral inflammation (Madeira et al., 2016). Melanocortin proteins upon receptor biding elevate the resolution of inflammation by reducing the amount of released pro-inflammatory cytokines and induce efferocytosis, the clearance of neutrophils by macrophages (Montero-Melendez et al., 2011). Besides the induction of citrullinating enzymes in neutrophils, LtxA has a strong toxic effect on leukocytes and induces NET formation in a dose-dependent manner. Additionally, the activity of neutrophil elastase (NE), a principal proteinase in bacterial defense, is exploited. Normally, NE is localized in the cytoplasm, however, upon LtxA exposure, neutrophil lysis is triggered, followed by the release of high amounts of elastase. As a consequence, human gingival epithelial cells and fibroblasts detach and die (Madeira et al., 2016; Hiyoshi et al., 2019).

Of note, *A. actinomycetemcomitans* activates many more neutrophil defense mechanisms, such as ROS production, the release of proteases and the already mentioned NET formation that can be considered a successful defense strategy in the absence of bacterial nuclease activity (Mikolai et al., 2021). Although, the coin has two sides, the overactivation of the above-mentioned mechanisms results in the destruction of the host tissues. Among other virulence factors, this pathogen produces a toxin called the Cytolethal Distending Toxin (CDT), which causes cell cycle arrest *in vitro* and *in vivo* as well as blocks proliferation of the target cells. These disease-promoting effects of CTD are noted towards the periodontal epithelial cells in the rat model (Ohara et al., 2011). Unfortunately, amoxicillin, azithromycin, and metronidazole show an attenuated efficiency against *A. actinomycetemcomitans*, while phagocytosis of the pathogen is only effective at a lower MOI (Multiplicity of Infection) (Ardila and Bedoya-García, 2020). Fortunately, when neutrophils are highly outnumbered by bacterial cells, internalized azithromycin significantly increases the phagocytic elimination efficiency of PMNs (Lai et al., 2015).

### Peptoanaerobacter stomatis and Filifactor alocis

*Peptoanaerobacter stomatis* is a newly characterized member of the destructive oral microbiome. In contrast to well-characterized periodontal pathogens, this one is Gram-positive (Sizova et al., 2015). Infection with this pathogen promotes migration of not just neutrophils, but also monocytes, which additionally strongly fuel inflammation, along with the vigorously induced granule content exocytosis (Vashishta et al., 2019). Furthermore, a significant induction of NET formation is observed upon neutrophil challenge with this pathogen (Armstrong et al., 2018). Moreover, *P. stomatis* is relatively resistant to phagocytosis, while ROS production is significantly increased. These types of defense mechanisms are a double-edged sword, because they simultaneously induce the degradation of the host gingival tissue and periodontitis progression (Flores et al., 2017).

Another Gram-positive member of the community is *Filifactor alocis* that shows an extraordinary resilience to oxidative stress as mentioned in Table 1. This provides a substantial colonization advantage over the host defense system and competing pathogens. Similar to other victorious pathogens, *F*. *alocis* manipulates the neutrophil immune responses. The analysis of global changes in the transcriptome of neutrophils challenged with *F*. *alocis* reveals strong effects on the PMNs. A delayed apoptosis is accompanied by a prolonged inflammatory response and activated migration through the MAPK cascade and the TNF-α signaling pathways (Miralda et al., 2020). Strikingly, *F. alocis* fails to induce NET formation, but doesn’t influence the *P. stomatis*-mediated NETosis. In contrast, an earlier challenge of neutrophils with *F. alocis* decreases NET formation triggered by PMA (Armstrong et al., 2018).

## Beyond Periodontitis

Recently, periodontal pathogens have started being linked to other, often life-threatening diseases, such as atherosclerosis, cardiovascular diseases or rheumatoid arthritis, as mentioned above (Baetta and Corsini, 2010; Steyers and Miller, 2014). *Campylobacter rectus* in addition to inhibit neutrophil elastase by ecotin, has been associated with hypertension (Pietropaoli et al., 2019; Thomas et al., 2020). *P. gingivalis* is the most abundant pathogen of all the detected species (Mougeot et al., 2017). Notwithstanding the fact that inflammation is an essential part of the defense of the immune system, in the case of chronic inflammation, the effect is the opposite. In the oral cavity, a dysbiotic and proinflammatory environment can accelerate the development of gum disease or can even lead to oral cancer. Accordingly, *P. gingivalis, F. nucleatum*, and *Treponema denticola* are the most frequently identified enriched species in patients with oral squamous cell carcinoma (OSCC) (Fitzsimonds et al., 2020). As indicated in Table 1, *T. denticola* can alter neutrophil migration as well as trigger a strong inflammatory response, mediated by the elevated expression of Oncostatin M (Jones et al., 2020). The identification of *P. gingivalis* and the secreted virulence factor (protease gingipain) in the brains of patients has been an important milestone in the research of Alzheimer’s disease. *In vivo* models demonstrated the pathogen’s ability to translocate from the oral cavity to the brain (Ilievski et al., 2018). By applying this knowledge, neurodegeneration can be reduced using specific gingipain inhibitors, which may be a promising treatment (Dominy et al., 2019). Of note, in rare cases, *F. nucleatum* was isolated from immunocompromised patients with pyogenic liver abscesses. However, this might be a slight contribution based on negligible case numbers (Collins and Diamond, 2021).

## Summary and Future Remarks

The scope of this article included anaerobic bacterial species. However, it has to be mentioned that also aerobic species (*Streptococcus* and *Staphylococcus*) avoid killing by neutrophils and can be found among other microorganisms leading to periodontal disease (Daniluk et al., 2006). As mentioned and summarized in Table 1, such pathogens start to be linked to other severe disorders and accelerated disease progressions. As a result of emerging detection methods, both the list of pathogens and the linked diseases will grow constantly, providing not just a better understanding, but also powerful diagnostic tools based on biomarkers in the long term (Han et al., 2021). Although it is almost impossible to list all the pathways and virulence factors, Table 1 presents the variety of bacterial adaptation mechanisms. The most abundant virulence factors are the proteases, but they are usually specific for the individual bacterial species. Therefore. such peculiarities hinder the development of new therapeutic approaches.

The shift of the oral microbiome and the emerging inflammation are the results of complex bacterial interactions and biofilm formation. Through the mentioned examples, it is demonstrated that pathogens develop an arsenal of functions to generate a favorable environment and to avoid killing by the most abundant immune cell type found in the oral cavity, the neutrophil. There are still many pathways to be discovered in the future and it cannot be ignored that this is not just a localized problem. Figure 1 illustrates that pathogens can use body fluids as highways to reach other parts of the body and promote inflammation. This might be the key information in some of the adverse pregnancy outcomes, where the placental microbiome shows an incredible resemblance to its assumed origin, the mouth (Fischer et al., 2019).

Interestingly, an investigation of ancient and traditional medications is still as beneficial as at the dawn of modern medicine. Daehwangmokdantang (DHMDT) is a polyherbal mixture known in ancient Korea. Another potential medicament can be an extract from a shrub called border forsythia (in Latin: *Forsythia x intermedia*) known in ancient China. Active lignans from its leaves or flowers have a similar anti-inflammatory effect through the MAPK/ERK pathway (Michalak et al., 2018). Therefore, the review of the underlying molecular mechanisms can bring us closer to efficient treatments (Máthé, 2020). The nuclear factor-κB (NF-κB) is the major transcription factor during inflammation that controls the expression of many pro-inflammatory factors, such as nitrite oxide (NO), prostaglandin (PG)E2, TNF-α and IL-1β (Li and Verma, 2002). Bacterial lipopolysaccharide (LPS) induces the translocation of NF-κB from the cytoplasm to the nucleus. In the presence of DHMDT, the process is inhibited and the expression of the mentioned pro-inflammatory substances is significantly reduced (Lee et al., 2017).

Statins are a class of widely used lipid-lowering medications that also have antimicrobial properties. A novel study by the group of dr Piotr Mydel (Kamińska et al., 2019), aiming to analyze statin effects on a dysbiotic oral microbiome *in vitro*, included different pathogens, such as *P. gingivalis, F. nucleatum*, *Actinomyces naeslundii*, *T. forsythia*, and *Streptococcus gordonii.* Results indicate high effectiveness against *P. gingivalis* without killing the commensal microbiota, which is a side effect of broad-spectrum antibiotics.

In summary, the future of periodontal medicine undoubtedly lies in a personalized approach as the microbial composition shows a huge variation between patients. An essential step along the way is the identification of contributing pathogens, their distinct biomarkers and the development of specific diagnostic tools (Van der Weijden et al., 2021).

## Author Contributions

ZP wrote and revised the manuscript, prepared figures, and tables. AP wrote and revised the manuscript. MS wrote, corrected, and revised the manuscript. JP corrected the manuscript. All authors contributed to the article and approved the submitted version.

## Conflict of Interest

The authors declare that the research was conducted in the absence of any commercial or financial relationships that could be construed as a potential conflict of interest.

## Publisher’s Note

All claims expressed in this article are solely those of the authors and do not necessarily represent those of their affiliated organizations, or those of the publisher, the editors and the reviewers. Any product that may be evaluated in this article, or claim that may be made by its manufacturer, is not guaranteed or endorsed by the publisher.

## Abbreviations

TLR: Toll-like receptor
MMPs: Matrix metalloproteinases
NET: Neutrophil extracellular trap
Msp: major outer sheath protein
eATP: extracellular adenosine triphosphate
NOD-like receptors: nucleotide-binding oligomerization domain-like receptors
HNP-1: Human Neutrophil Peptide-1
hDFSCs: Human dental follicle stem cells
PMNs: polymorphonuclear leukocytes
GSDs: Glycogen storage diseases
S-layer: surface layer
MAPK: mitogen-activated protein kinase
MK2: MAPK-activated protein kinase 2
ROS: reactive oxygen species
IL: interleukin
SCFAs: short-chain fatty acids
Spp.: Species
LtxA: leukotoxin A
NE: Neutrophil elastase
CDT: cytolethal distending toxin
MOI: Multiplicity of infection
TNFα: tumor necrosis factor alpha
OSCC: oral squamous cell carcinoma
NF-κB: nuclear factor-κB
NO: nitrite oxide
PG: prostaglandin
LPS: lipopolysaccharide
DHMDT: Daehwangmokdantang
ERK: extracellular signal-regulated kinases.
